# Effects of Virtual Nature Embodiment on Compassion, Empathy, Nature Connectedness, and Symptom Burden in Patients With Psychosis or Depression: An Explorative Clinical Study

**DOI:** 10.2196/74337

**Published:** 2026-03-24

**Authors:** Alva Lütt, Kristina Lütgens, Christiane Montag, Stefan Gutwinski, Georg Felix Reuth, Steve Nebel, Felix Bermpohl, Pia Spangenberger

**Affiliations:** 1 Department of Psychiatry and Neurosciences, Charité Campus Mitte, Charité – Universitätsmedizin Berlin Berlin Germany; 2 Department of Psychiatry and Psychotherapy, Charité at St. Hedwig Hospital, Charité – Universitätsmedizin Berlin Berlin Germany; 3 German Center for Mental Health (DZPG), partner site Berlin Berlin Germany; 4 Berlin Institute of Health at Charité - Universitätsmedizin Berlin, BIH Biomedical Innovation Academy, BIH Charité Junior Digital Clinician Scientist Program Berlin Germany; 5 Department of Psychology Humboldt-Universität zu Berlin Berlin Germany; 6 Department of Education University of Potsdam Potsdam Germany

**Keywords:** immersive virtual reality, nature connectedness, psychosis, depression, compassion, empathy, symptom burden, virtual body ownership

## Abstract

**Background:**

Nature experiences may have a positive impact on mental health. Innovative alternatives, such as immersive virtual reality (iVR), can have similar effects. In previous studies on embodying a tree in virtual reality, connectedness to nature has been induced in healthy participants and shown to be influenced by compassion. Compassion and empathy, however, can be altered in psychiatric disorders, leading to impaired relationships with fellow human beings. The potential effect of nature experiences in iVR on compassion, empathy, and nature connectedness in mental health disorders has not yet been investigated.

**Objective:**

This study aims to examine the development of nature connectedness, compassion, empathy, and individual symptom load in patients with depression (n=20), schizophrenia (n=20), and healthy controls (n=20), measured pre and post 1 session of virtual embodying a rainforest tree.

**Methods:**

We conducted an explorative clinical trial, comparing 3 groups (depression, schizophrenia, and healthy control), using repeated measures ANOVA and multiple regression analysis. Effect sizes (η_p_^2^) and 95% CIs were reported where applicable. We assessed the impact of iVR-exposure on nature connectedness, empathy, compassion, individual symptoms, spirituality, cybersickness, presence, and virtual body ownership. Electrodermal activity was measured to capture physiological correlates of emotional arousal.

**Results:**

Individual symptom load decreased significantly through the experience of embodying a growing tree in iVR in both patient groups (*F*_1,38_=40.93, η_p_^2^=0.52, 95% CI 0.29-0.67; *P*<.001). All groups benefited equally from the iVR experience regarding a change in nature connectedness (*F*_1,57_=100.12, η_p_^2^=0.637, 95% CI 0.48-0.74; *P*<.001) and compassion (*F*_1,57_=12.86, η_p_^2^=0.18, 95% CI 0.04-0.36; *P*<.001). The change in empathy did not differ significantly between the 3 groups. The analysis of electrodermal activity during iVR showed significantly higher nonspecific skin conductance response numbers than at the beginning of the iVR (2-tailed *t*_53_=4.40, mean difference=2.38, 95% CI 1.30-3.46; *P*<.001). Changes in compassion were predicted by spirituality (*β*=–0.35, 2-tailed *t*_59_=–2.89, η_p_^2^=0.13, 95% CI 0.01-0.30; *P*=.005) and virtual body ownership (*β*=0.29, 2-tailed *t*_59_=2.15, η_p_^2^=0.08, 95% CI 0.00-0.23; *P*=.04) but not by the feeling of presence.

**Conclusions:**

Our results indicate that virtually embodying a tree seems to have a positive effect on individual symptoms. Patients with depression and schizophrenia were able to receive an iVR-based intervention and benefited from the experience. Our results provide a deeper understanding of the phenomenon of virtual body ownership of nature, a specific affordance of iVR, thereby laying the groundwork for future innovative body psychotherapy interventions for individuals with mental health disorders.

**Trial Registration:**

ClinicalTrials.gov NCT06446856; https://tinyurl.com/nhjar26p

## Introduction

### Background

Positive effects of nature experiences on mental health have been shown by numerous studies and meta-analyses, especially in depressive and anxiety disorders [1-3]. However, a recent systematic review by Paredes-Céspedes et al [4] from 2024 concluded that outcomes of nature-based therapy studies were inconclusive in regard to definitive effects on stress, anxiety, or depression, for example, due to varying interventions, sample sizes, and methods, thereby highlighting the need for further studies on this topic [4]. Regarding the potential therapeutic effects of experiencing nature, the practicability of potential interventions in everyday clinical routines for patients, which sometimes take place far away from nature (eg, in hospitals), has to be considered. Additionally, urbanization means that fewer and fewer people are able to experience nature directly [5].

Virtual reality (VR)–based applications are becoming increasingly important in the development of innovative, easily accessible, therapeutic procedures in mental health care [6,7]. Patients can immerse themselves in various computer-generated environments with the help of a head-mounted display (HMD), ranging from, for example, exposure scenarios for anxiety disorders or substance use disorders to avatar-based interventions for psychotic disorders [6]. Exploring these 3D scenarios, a feeling of “presence,” of being in this virtual world, can arise, the subjective correlate of “immersion” as the computer system’s capacity to deliver a vivid experience [8]. Related to the sense of presence, “embodiment” is another key concept in immersive virtual reality (iVR), with one of its subcomponents being “virtual body ownership” [9]. Although an experience in iVR cannot replace real experiences in nature [10], a recent study in healthy volunteers compared specific emotions, presence, and immersion in iVR with real natural environments and found that only some aesthetic emotions, state anxiety, and presence, but not immersion, were significantly more intense in real situations than in iVR environments [11]. Studies show that the level of immersion and realism is highly relevant for affective responses to nature scenarios, underlining the importance of iVR over, for example, videos [12]. Especially, virtual body ownership seems to be a promising approach to foster affective and cognitive processes relevant to the feeling of nature connectedness [13,14]. Hence, it is not surprising that interest in iVR-based nature exposure is also increasing in the field of mental health.

Ilioudi et al [15] described the patient experiences of 20 patients with bipolar and unipolar depression who used an iVR calm room with relaxing nature scenarios in an inpatient psychiatric setting. Qualitative interviews showed an increase in awareness, calmness, and well-being as well as patients’ gratefulness about these nonpharmacological alternatives for anxiety relief [15].

Another pilot study on the feasibility and acceptability of an iVR relaxation intervention in an acute psychiatric setting showed a statistically significant increase in relaxation, happiness, and connectedness to nature and a decrease in stress, anxiety, and sadness, all measured by visual analog scales (VASs) [16]. Even effects on violence were shown—according to the authors, violent incidents and restrictive practices decreased during the implementation of this iVR-application. The intervention consisted of 1 session, maximum 1 hour, with several possible scenarios open for exploration (eg, a beach and scuba diving with dolphins [16]).

Despite these promising examples, the induction of affective processes and their effects on different psychiatric disorders of such iVR-based nature experiences remain relatively unexplored. In a preceding study with healthy students embodying a tree in a virtual rain forest, previous studies could show that compassion is a crucial influencing factor for the development of nature connectedness [17]. However, deficits in compassion and empathy are described in several psychiatric disorders, whereas patients with schizophrenia, examined with the Multifaceted Empathy Test (MET) and Interpersonal Reactivity Index, showed impairments in cognitive empathy but not emotional empathy [18]; patients with persistent or recurrent depression completing the same tests had deficits in emotional but not cognitive empathy [19]. The importance of these abilities for psychosocial functioning, or on the other side, isolation and social withdrawal is obvious [18,19]. Whether these impairments also influence connectedness to nature in patients with psychotic or depressive disorders remains unclear.

In addition to the development of nature connectedness, iVR-based interventions, especially the experience of virtually embodying a tree, could lead to an enhancement of body experiences, which are also the focus of body psychotherapy interventions [20]. The question arises whether a single iVR intervention is already associated with changes in affect and symptom load, thereby indicating a potential therapeutic effectiveness of such interventions, which could then be investigated in further studies.

### Objective of the Study

This explorative study aims to examine the effect of 1 session of an iVR-based nature experience in 2 different patient groups with highly prevalent serious mental illnesses, patients with depressive disorders, and patients with schizophrenia, compared with a healthy control (HC) group. We test our hypotheses that patients with a depressive or psychotic disorder and HCs show differential effects of the iVR intervention on (1) nature connectedness, (2) empathy, and (3) compassion. In addition, we hypothesize that (4) patients with psychotic or depressive disorder have a lower symptom burden after the iVR experience than before the iVR experience. On an explorative basis, we test the hypothesis that (5) the measurement of electrodermal activity (EDA) before, during, and after the iVR application allows emotional arousal to be objectified. Additionally, we test the explorative hypothesis (H6) that spirituality, presence and virtual body ownership have an influence on nature connectedness, empathy and compassion in all 3 groups.

## Methods

### Study Design

This clinical trial study aimed to evaluate symptom load, empathy, compassion, and nature connectedness before and after an iVR experience in patients with depressive disorder, patients with schizophrenia, and HC participants (participants between 18 and 65 years of age).

**Figure 1:**
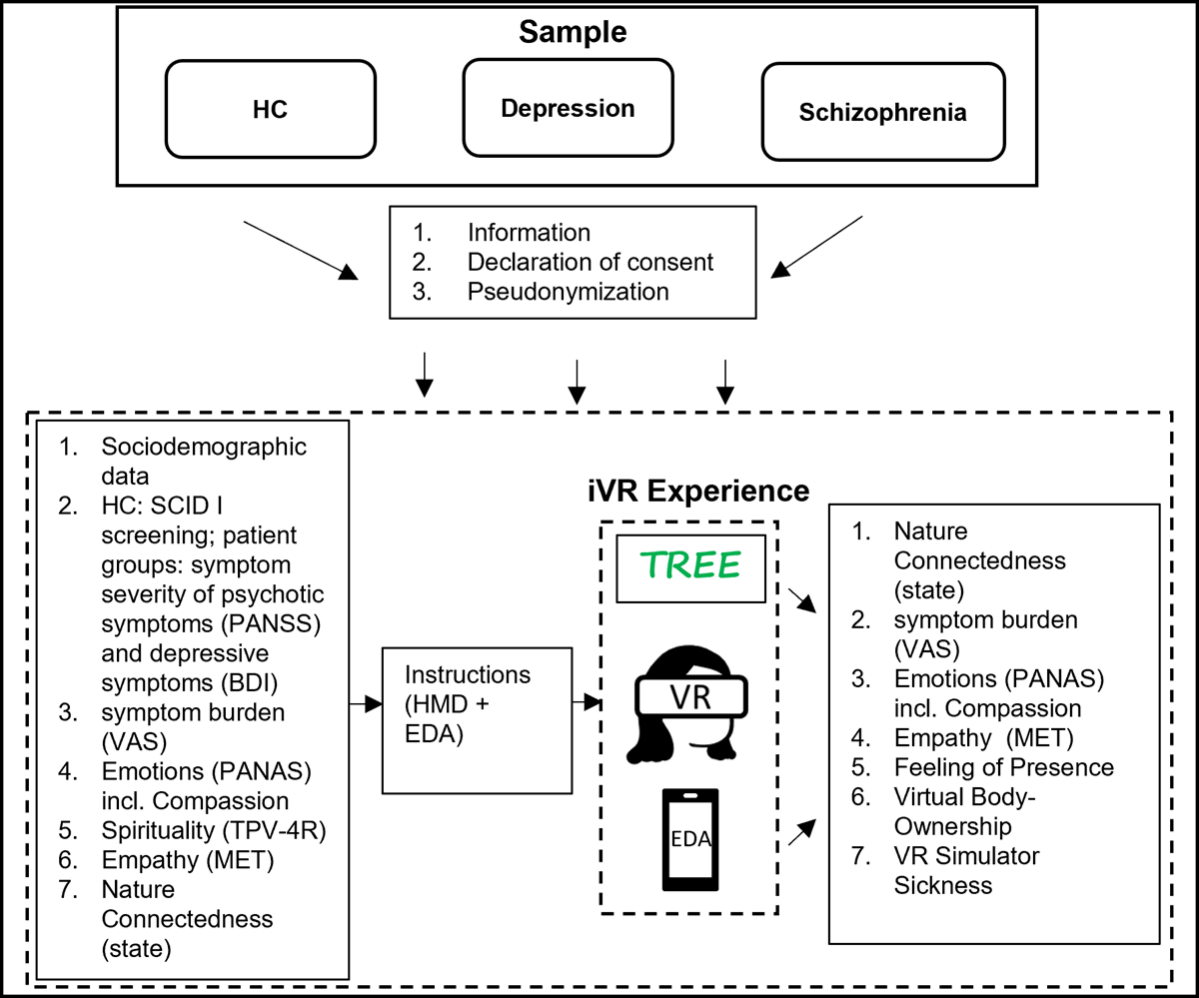
Study Design.

The study was designed as follows. Before participating in the study, participants were informed about the nature of the experiment and declared consent (Figure 1). Symptom severity was assessed with the Beck Depression Inventory (BDI) for depressive symptoms and the Positive and Negative Syndrome Scale (PANSS) for psychotic symptoms. HCs were screened with the Structured Clinical Interview for *DSM-IV* (*Diagnostic and Statistical Manual of Mental Disorders* [Fourth Edition]; SCID I). Being a pre-post intervention, participants filled out an online questionnaire before the VR-exposure, containing questions on sociodemographics, symptom burden perceived in the moment with a VAS, as well as perceived nature connectedness (state), spirituality, and emotions, including compassion (Positive and Negative Affect Schedule [PANAS]) and empathy (MET). After filling out the questionnaire, participants put on the VR headset and were equipped with an EDA measurement device attached to the arm and fingers. After experiencing the VR application for 4 minutes and 20 seconds, participants filled out the post-VR questionnaire, containing items on symptom burden (VAS), nature connectedness (state), emotions including compassion (PANAS), feeling of presence, virtual body ownership, VR simulator sickness, and empathy (MET).

### Setting

The experimental setting included a notebook for questionnaire administration and the VR hardware (refer to Material section), arranged in a setting that provided sufficient space for participants. Data collection took place at the Psychiatric University Hospital Charité at St. Hedwig Hospital, Berlin, Germany, over a 4-week period (May 23-June 19, 2024), during which participants were recruited, and data were assessed. Participants were reimbursed with €15 (approximately US $17).

### Material

**Figure 2:**
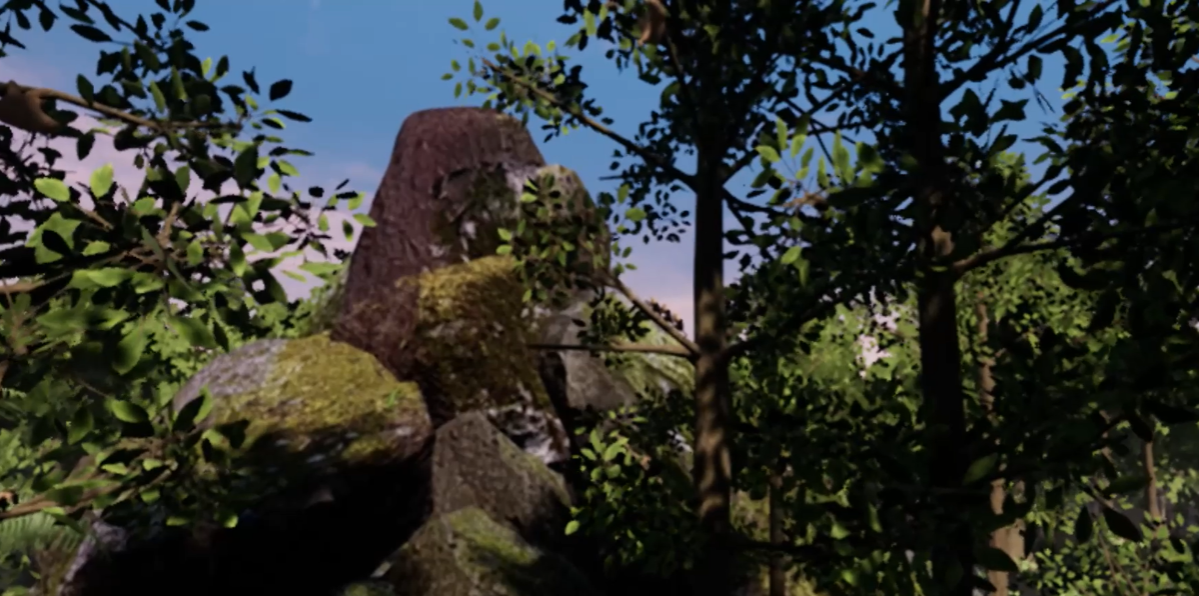
Own Screenshot of the VR-application Tree purchased from VIVEPORT developed by New Reality Co. (http://newreality.co) [21, 22]. Used under quotation exception for research purposes.

The iVR application “Tree” was used, developed by a group of researchers from the Massachusetts Institute of Technology and filmmakers [21,22]. This application has been used in previous studies to examine its impact on nature connectedness within healthy individuals, observing a positive effect on nature connectedness [13,14,17]. In the application, users explore the growth of a rainforest tree, starting from the viewpoint of a seed under the earth to a fully-grown tree reaching the sky (refer to Figure 2 for a screenshot from the VR application). The illusion of virtual body ownership was induced by synchronous movements of, for example, the arms of participants and the corresponding branch of the tree. While users embody the growing tree, they can watch various animals (eg, ants, birds, and monkeys) around them, as well as other trees in the jungle. In the original version, a forest fire occurs at the end of the experience. However, in this study, we used only the nonthreatening part of the iVR experience, stopping the application after 4 minutes and 20 seconds. The VR sessions were conducted by a psychiatrist (AL) and a second trained experimenter (KL) with experience in VR application and treatment of patients with acute psychiatric conditions. The hardware used for the VR exposure consisted of an HMD (HTC Vive Focus 3, including controllers, field of view 120°, resolution of 2448×2448 pixels per eye, 6DoF, 90Hz refresh rate), a notebook, an Android smartphone, and an EDA device. Patients were standing throughout the exposure and were able to move. The HMD was connected to an Alienware notebook during the entire experiment (Intel Core i9-8 8950HK processor, 16 GB RAM, NVIDIA GeForce GTX 1080, Windows 10 Home). The smartphone was a Samsung S10e running on Android 12, connected to the Mindfield eSense Skin Response Meter (recording at 10Hz).

### Participants

Participants were inpatients or outpatients and HCs recruited via an open call (purposive sampling) at the Psychiatric University Hospital Charité at St. Hedwig Hospital, Berlin, Germany. Interested parties were invited to a brief information session (approximately 5-10 minutes), during which it was also assessed whether the inclusion criteria were met. The following inclusion and exclusion criteria (Textbox 1) were binding for participating in the study.

**Inclusion and exclusion criteria.**

**Inclusion criteria**

- Age: 18 to 65 years
- Inpatients or outpatients of the Psychiatric University Hospital Charité at St. Hedwig Hospital
- Diagnosis of schizophrenia (F20; schizophrenia) or unipolar depressive disorder according to the *ICD-10* (*International Statistical Classification of Diseases, Tenth Revision*; F32, F33; depression) or healthy controls between 18 and 65 years
- Ability to provide written informed consent after receiving study information

**Exclusion criteria**

- Acute suicidality or endangerment of others
- Primary eating disorder requiring treatment
- Acute dermatological condition affecting the hands that could interfere with electrodermal activity measurement
- Healthy control group: Diagnosis of a psychiatric or psychosomatic disorder; additional exclusion criteria corresponding to those of the patient group

### Assessments

All participants were exposed to the virtual body ownership experience as described in the previous sections. Hypotheses of this study can be found in the Introduction section. Outcomes and methods of assessment are described in detail in the Data Sources section.

### Data Sources

#### Nature Connectedness

Measurement of nature connectedness as a state variable with the “Inclusion of Nature in Self” (INS) scale [23] before and after iVR exposure, based on the German version by Spangenberger et al [13]. The item consisted of a slider based on percentages (0%=zero overlap of “I” and “nature” and 100%=total overlap). Calculating the test-retest reliability revealed a satisfying Pearson correlation of *r*=0.64.

#### Empathy

Evaluation of cognitive empathy and emotional empathy explicit with the MET [24], before and after the iVR exposure. Questions on emotional empathy implicit (levels of arousal) were excluded. The questionnaire was divided into 2 equal parts, which the patients completed in pseudorandomized order before and after the iVR exposure. Internal reliability measures were conducted for both pseudorandomized orders and both subscales. The cognitive empathy scale revealed an acceptable internal reliability for both pseudorandomized orders (Order A: pre iVR McDonald ω=0.64, post iVR McDonald ω=0.74; and Order B: pre iVR McDonald ω=0.75, post iVR McDonald ω=0.73). The emotional empathy scale revealed an excellent internal reliability for both pseudorandomized orders (Order A: pre iVR McDonald ω=0.97, post VR McDonald ω=0.97; and Order B: pre iVR McDonald ω=0.95, post iVR McDonald ω=0.97).

#### Compassion

Evaluation of compassion (state compassion) pre and post the iVR exposure with the PANAS and 5 integrated items measuring compassion based on Pfattheicher et al [25]. The compassion scale revealed a good internal reliability for pre (McDonald ω=0.85) and post iVR exposure (McDonald ω=0.85).

#### Symptom Burden

Symptom burden was assessed with the VAS (0-10) measuring the burden of the individual 3 main symptoms, previously specified during assessment of symptom severity (PANSS based on Kay et al [26]) and BDI based on Beck et al [27]. Calculating the test-retest, the VAS reliability revealed a satisfying Pearson correlation of *r*=0.62.

#### Explorative EDA

EDA was analyzed using the counted number of nonspecific skin conductance responses (NS-SCRs) provided by the Mindfield eSense Skin Response device during iVR exposure.

#### Structured Clinical Interview for DSM-IV (SCID I)

The screening with SCID I was conducted before the iVR exposure to screen for psychiatric symptoms in the HC group based on First and Gibbon [28]. The SCID I revealed an excellent internal reliability (McDonald ω=0.90; item 3 excluded due to an SD 0).

#### VR Simulator Sickness

Motion sickness as a potential side effect of iVR, measured with the Virtual Reality Sickness Questionnaire based on Kim et al [29], after exposure. This questionnaire revealed an acceptable internal reliability (McDonald ω=0.70).

### Definition of Covariates

*Embodiment* was assessed as the feeling of virtual body ownership with an adapted and translated subscale of the Embodiment scale by Spangenberger et al [13], based on Ahn et al [30] and Slater et al [31], consisting of 5 items. This questionnaire revealed an excellent internal reliability (McDonald ω=0.95).

*Spirituality* was assessed using the 4-item short version of the questionnaire “Transpersonales Vertrauen” based on Hampel et al [32] before iVR exposure. This questionnaire revealed a good internal reliability (McDonald ω=0.81).

Feeling of presence in iVR: Scale according to Ahn et al [30], adapted from the “Spatial Presence scale” by Bailenson et al [33], German translation by Spangenberger et al [13], adapted to the virtual Amazon forest area after exposure. The internal reliability of this questionnaire was acceptable (McDonald ω=0.79).

*PANSS* was based on the study by Kay et al [26]. Furthermore, 5 subscores according to van der Gaag et al [34] were calculated to better differentiate between positive symptoms, negative symptoms, disorganization, excitement, and emotional distress. The PANSS total internal reliability was excellent (McDonald ω=0.93).

*BDI* was based on the study by Beck et al [27]. The BDI revealed a good internal reliability (McDonald ω=0.87).

### Study Size

Due to the exploratory nature of this study and the lack of comparable studies, the number of cases is not based on a sample size calculation but based on a consideration of feasibility (given the technically complex preparation and implementation), and is intended to provide a basis for subsequent follow-up studies. Effect sizes in the 3 groups could be used in a follow-up study for power analyses and thus for calculating sample size.

### Data Analysis

All outcome variables were checked for outliers and extreme values before conducting the analyses. The assumptions for analysis of variance were also examined [35]. The results are presented in the Multimedia Appendix 1.

Baseline characteristics between the 3 groups were compared using either the chi-square test or, if cell counts were less than 5, the Fisher exact test.

To analyze hypothesis 1 (differences in nature connectedness), hypothesis 2 (differences in cognitive empathy and emotional empathy explicit), hypothesis 3 (differences in compassion), and hypothesis 4 (differences in mean VAS scores pre- vs post-iVR exposure), we calculated a repeated measures ANOVA and post hoc tests (Tukey [36]). Additionally, we conducted ANCOVA with depressive (BDI sum score) and psychotic symptom severity (PANSS subscales according to the 5-factor solution by van der Gaag et al [34]) as covariates and delta VAS as the dependent variable.

To test for physical emotional arousal (hypothesis 5), EDA was recorded throughout the experience using the eSense skin-response device from Mindfield, capturing data in µS at a 5 Hz sampling rate. Then, 2 electrodes were attached to the participants’ index and middle fingers on the palm side and connected by cable to a smartphone that used the eSense App to measure EDA through skin conductance and NS-SCR. The number of NS-SCRs at the end of the first full minute was set as the baseline. Paired-samples *t* tests were conducted to examine the difference between the mean of the number of NS-SCRs per minute during the iVR and the baseline.

We tested the direct effects postulated in hypothesis 6 with 4 multiple linear regression analyses, examining the influence of each independent variable as a predictor (spirituality, presence, and virtual body ownership) and the combination of them on the dependent variables (nature connectedness, empathy, and compassion).

### Ethical Considerations

The authors assert that all procedures contributing to this work comply with the ethical standards of the relevant national and institutional committees on human experimentation and with the Helsinki Declaration of 1975, as revised in 2008. The study was approved by the Ethics Committee of Charité - Universitätsmedizin Berlin Institutional Review Board (EA2/04/24; April 26, 2024) and preregistered on ClinicalTrials.gov. All participants gave written informed consent after a previous verbal explanatory discussion and the opportunity to ask questions. They received compensation of €15 (approximately US $17) for their participation. All study data were pseudonymized by sequential numbering (without using initials or dates of birth), and no identification through images or supplementary material is possible.

## Results

### Descriptive Statistics

Baseline descriptive characteristics of the study participants are displayed in Table 1. One participant stopped the application early due to general discomfort. All other participants completed the full VR application. Mean Virtual Reality Sickness Questionnaire scores were 1.40 (SD 0.301, range 1.00-2.22) for the patient groups, including 3 outliers, and 1.26 (SD 0.173, range 1.00-1.56) for the HC group. Patients showed a mean value of the virtual body ownership items of 4.36 (SD 1.36, range 2.00-7.00), and HCs showed a mean value of 5.35 (SD 1.37, range 1.60-7.00). The presence questionnaire post VR resulted in a mean value of 3.65 (SD 0.708, range 2.40-5.00) for patients and a mean value of 3.60 (SD 0.676, range 2.40-5.00) for HCs.

**Table 1:** Sample characteristics.

Characteristics and categories				HC^a^ (n=20)		Schizophrenia (n=20)		Depression (n=20)		Statistical value, *F* test (*df*) or chi-square (*df*)		*P* value	
**Demographics**													
	Age (y), mean (SD)		37.75 (9.94)		43.75 (10.36)		45.05 (15.42)		2.05 (2,57)^b^		.14		
	**Sex, n (%)**								0.93 (2)^c^, N=60		.63		
		Male	10 (50)		12 (60)		9 (45)						
		Female	10 (50)		8 (40)		11 (55)						
	**Treatment setting, n (%)**								—^d^		.43^e^		
		In-patient	—		13 (65)		12 (60)						
		Day clinic	—		1 (5)		4 (20)						
		Out-patient	—		6 (30)		4 (20)						
	Years of education, mean (SD)		18.90 (3.39)		15.80 (6.38)		14.60 (6.11)		9.86 (2)^c,f^, N=60		.007^f^		
	BMI (kg/m^2^), mean (SD)		24.05 (5.08)		28.24 (7.59)		27.62 (5.45)		6.83 (2)^c,f^, N=60		.03^f^		
	**Living situation, n (%)**								—		<.001^e^		
		Living alone	7 (35)		17 (85)		4 (20)						
		Parents	1 (5)		1 (5)		1 (5)						
		Partner	5 (25)		0 (0)		8 (40)						
		Sibling or other relatives	4 (20)		0 (0)		2 (10)						
		Shared apartment or residential home	3 (15)		2 (10)		5 (25)						
	Netto income after deduction of fix expenses, mean (SD)		1080.00 (616.95)		503.65 (424.77)		703.50 (405.08)		12.56 (2)^c,f^, N=60		.002^f^		
	**Marriage status^g^, n (%)**												
		Single	12 (60)		18 (90)		10 (50)		—		.02^e^		
		Married	4 (20)		0 (0)		6 (30)		—		.03^e^		
		Divorced	2 (10)		2 (10)		3 (15)		—		>.99^e^		
		Living separately	1 (5)		0 (0)		0 (0)		—		>.99^e^		
		Widow or widower	0 (0)		0 (0)		0 (0)		—		—		
		Monogamous	1 (5)		0 (0)		2 (10)		—		.77^e^		
		Polygamous	1 (5)		0 (0)		0 (0)		—		>.99^e^		
	**Comorbidities, n (%)**								10.58 (2)^c^, N=60		.005		
		No	15 (75)		8 (40)		5 (25)						
		Yes	5 (25)		12 (60)		15 (75)						
	**Somatic comorbidity^h^, n (%)**								5.76 (2)^c^, N=60		.06		
		No	15 (75)		9 (45)		8 (40)						
		Yes	5 (25)		11 (55)		12 (60)						
	**Psychiatric comorbidites, n (%)**								—		.004^e^		
		No	20 (100)		19 (95)		13 (65)						
		Yes	0 (0)		1 (5)		7 (35)						
**Clinical baseline data**													
	**PANSS^i^ score, mean (SD)**												
		Total	—		98.75 (31.67)		78.55 (19.33)		5.93 (1,31.43)^b,j^		.02^j^		
		Positive symptoms	—		18.85 (7.57)		10.4 (3.05)		21.44 (1,25.013)^b,j^		<.001^j^		
		Negative symptoms	—		18.7 (8.09)		17.5 (7.86)		0.17 (1)^c,f^, N=40		.68^f^		
		Disorganization	—		22.1 (7.31)		14.35 (3.72)		17.85 (1,28.207)^b,j^		<.001^j^		
		Excitement	—		18.05 (6.99)		13.8 (3.9)		5.64 (1,29.785)^b,j^		.02^j^		
		Emotional distress	—		21.05 (7.48)		22.5 (5.33)		0.50 (1,38)^b^		.48		
	BDI^k^ score, mean (SD)		—		18.45 (8.62)		27.3 (9.83)		9.16 (1,38)^b^		.004		

^a^HC: healthy control.

^b^*F* test.

^c^Chi-square value.

^d^Not applicable.

^e^Fisher exact test.

^f^Kruskal-Wallis test.

^g^Multichoice option→percentage of n=20.

^h^Somatic comorbidity refers to any diagnosis of a somatic disorder.

^i^PANSS: Positive and Negative Syndrome Scale.

^j^Welch-ANOVA.

^k^BDI: Beck Depression Inventory.

### Hypothesis Outcomes

#### Hypothesis 1: Patients With a Depressive or Psychotic Disorder and HCs Show Differential Effects of the iVR Intervention on Nature Connectedness

We found a strong increase in nature connectedness on comparing before and after experiences for all groups. Nature connectedness from before to after the iVR experience, INS delta, increased for participants with depression (mean 28.85, SD 26.55), psychotic disorder (mean 32.65, SD 25.86), and HCs (mean 30.70, SD 18.00). The repeated measures ANOVA showed a significant effect of time on changes in nature connectedness with a narrow CI, suggesting a high degree of precision in the effect size estimate (*F*_1,57_=100.12, η_p_^2^=0.637, 95% CI 0.48-0.74; *P*<.001) but no significant interaction of group and time (*F*_2,57_=0.128, η_p_^2^=0.004, 95% CI 0.00-0.11; *P*=.88). Hypothesis 1 has to be rejected.

#### Hypothesis 2: Patients With a Depressive or Psychotic Disorder and HCs Show Differential Effects of the iVR Intervention on Empathy

The repeated measures ANOVA showed neither significant effects of time on emotional empathy explicit (*F*_1,57_=0.03, η_p_^2^=0.001, 95% CI 0.00-0.06; *P*=.86) or cognitive empathy (*F*_1,57_=0.77, η_p_^2^=0.013, 95% CI 0.0-0.1; *P*=.38) for the 3 groups, nor significant interaction of group and time (emotional empathy explicit: *F*_2,57_=0.68, η_p_^2^=0.023, 95% CI 0.00-0.22; *P*=.51; and cognitive empathy: *F*_2,57_=0.54, η_p_^2^=0.018, 95% CI 0.0-0.2; *P*=.59). Hypothesis 2 has to be rejected.

#### Hypothesis 3: Patients With a Depressive or Psychotic Disorder and HCs Show Differential Effects of the iVR Intervention on Compassion

The repeated measures ANOVA revealed a significant effect of time (*F*_1,57_=12.86, η_p_^2^=0.18, 95% CI 0.04-0.36; *P*<.001) for the change in compassion but no significant interaction of group×time (*F*_2,57_=1.48, η_p_^2^=0.05, 95% CI 0.0-0.3; *P*=.24). Hence, compassion increased for all participants after iVR exposure but did not differ significantly between groups. Calculations were repeated after the removal of 3 outliers, showing no significant differences. Hypothesis 3 has to be rejected.

#### Hypothesis 4: Patients With Psychotic or Depressive Disorder Have a Lower Symptom Burden After the VR Experience Than Before the iVR Experience

**Figure 3:**
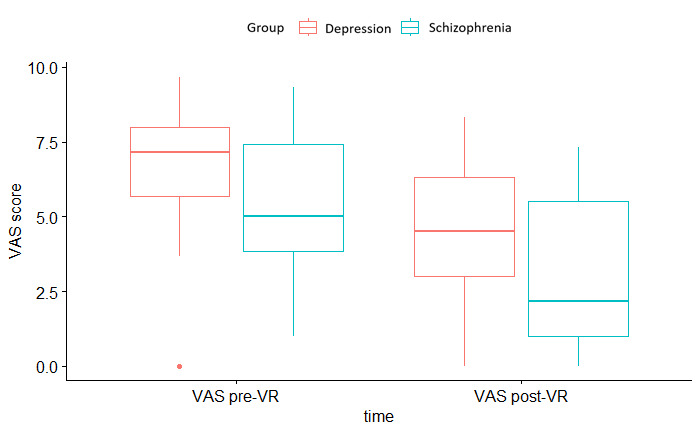
Boxplot of pre- and post-VR VAS measures in both patient groups.

Repeated measures ANOVA revealed a significant effect of time—both groups, patients with psychotic and depressive disorders, showed significantly fewer symptoms after iVR exposure (*F*_1,38_=40.93, η_p_^2^=0.52, 95% CI 0.29-0.67; *P*<.001), indicating a large and precisely estimated effect. There was also a significant effect of group (*F*_1,38_=4.68, η_p_^2^=0.11, 95% CI 0.000-0.314; *P*=.04), indicating significant differences between the groups, but no interaction effect of group and time (*F*_1,38_=0.021, η_p_^2^=0.001, 95% CI 0.000-0.074; *P*=.89). Patients diagnosed with depression had a mean VAS score of 6.58 (SD 2.21) before and a mean VAS score of 4.42 (SD 2.50) after the iVR experience. Patients diagnosed with schizophrenia had a mean VAS score of 5.18 (SD 2.31) before and 2.92 (SD 2.52) after iVR. Hypothesis 4 was confirmed. Figure 3 illustrates a boxplot of pre- and post-VR VAS measures as a measure of symptom burden in both patient groups.

To further explore the influence of the iVR intervention on the change in symptom load of patients with psychosis or depression measured by VAS of the 3 individual main symptoms, we performed an ANCOVA controlling for the covariates, psychotic and depressive symptom severity as assessed with the PANSS, and the BDI before the iVR experience.

The overall model was not statistically significant (*F*_7,32_=1.44, *R*^2^=0.239, 95% CI 0.000-0.364; *P*=.23). Furthermore, 2 covariates, however, emerged as significant—PANSS factor excitement (*β*=–0.198, η_p_^2^=0.127, 95% CI 0.000-0.353, ω^2^=0.086; *P*=.03) and PANSS factor negative symptoms (*β*=0.165, η_p_^2^=0.063, 95% CI 0.00-0.27, ω^2^=0.096; *P*=.03). This suggests that higher levels of excitement symptoms are rather associated with a decrease in change of symptom load (delta VAS), whereas higher levels of negative symptoms are rather associated with an increase in change of symptom load (delta VAS).

#### Hypothesis 5: The Measurement of EDA Before, During, and After the iVR Application Allows Emotional Arousal to Be Objectified

In total, 59 participants were tested for emotional arousal. In 1 case, the EDA device did not work properly because it lost connectivity during the intervention after 3 minutes. In 4 cases, the EDA device recorded no change over time, hinting at a bad connection of the sensor. The average number of counted NS-SCRs at the end of the first full minute in iVR of the remaining 54 participants was interpreted as a baseline (mean 6.92, SD 3.65, 95% CI 5.93-7.90). A relatively low level of physical arousal (mean of the average number of NS-SCRs per minute) during the experience was measured over time (mean 9.30, SD 4.27, 95% CI 8.13-10.05). A paired-samples *t* test was conducted to examine the difference between the average number of counted NS-SCRs at baseline (point 1) and the mean of NS-SCRs during iVR (point 2). The analysis showed that the difference between point 1 (baseline) and point 2 (during) was statistically significant but with a wide CI (2-tailed t_53_=4.40, *P*<.001, mean difference=2.38, 95% CI 1.30-3.46). EDA before the iVR session was not assessed. Therefore, hypothesis 5 was partly confirmed, as analysis of EDA during iVR showed significantly higher NS-SCR numbers than at the beginning of the iVR application.

**Figure 4:**
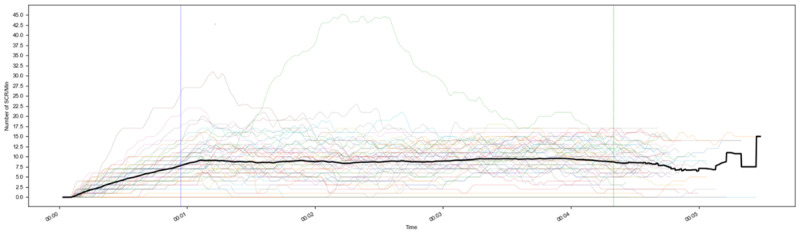
Number of NS-SCRs per minute.

In Figure 4, the black line presents the average number of NS-SCRs per minute of all participants, whereas the colored lines are the individual numbers of NS-SCRs per minute. One point in time is marked as the first event when breaking through the soil. The second point in time is marked as an event by means of the end of the scenario.

#### Hypothesis 6: Spirituality of the Participants, Presence, and Virtual Body Ownership in iVR Have an Influence on Intervention-Induced Changes in Nature Connectedness, Empathy, and Compassion in all 3 Groups

The multiple linear regression analyses revealed that the combination of the 3 predictors (spirituality, presence, and virtual body ownership) did not explain changes in nature connectedness (state; *R*^2^=0.04, *F*_3,56_=0.72, 95% CI 0.00-0.13; *P*=.55).

The multiple linear regression analysis also revealed that the combination of the 3 predictors (spirituality, presence, and virtual body ownership) did not explain changes in emotional empathy (state; *R*^2^=0.06, *F*_3,56_=1.21, 95% CI 0.00-0.18; *P*=.31).

Similar results were observed for changes in cognitive empathy. The test revealed that the combination of the 3 predictors (spirituality, presence, and virtual body ownership) did not explain changes in cognitive empathy (state; *R*^2^=0.02, *F*_3,56_=0.382, 95% CI 0.0-0.09; *P*=.77).

**Figure 5:**
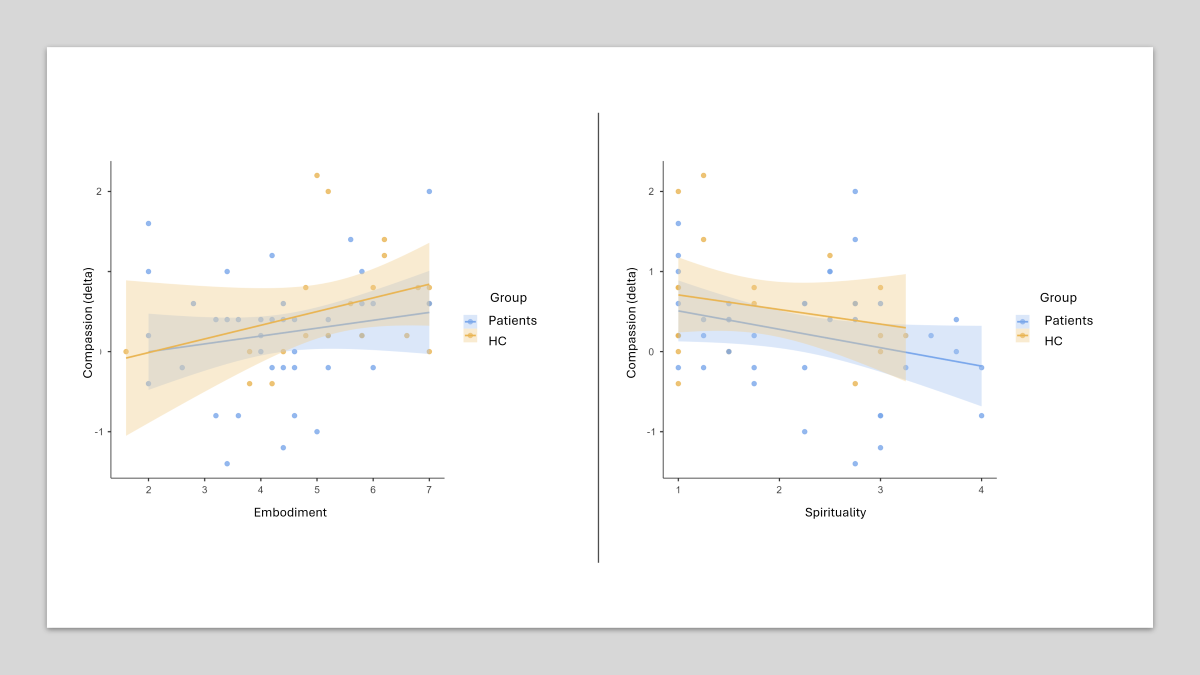
Prediction of changes in the dependent variable compassion by the independent variables spirituality and embodiment.

For compassion, the test revealed that the combination of the 3 predictors (spirituality, presence, and virtual body ownership) explained 20.2% of the variance in participants’ change in compassion (state; *R*^2^=0.20, *F*_3,56_=4.73, 95% CI 0.04-0.34; *P*=.005). Whereas the predictor variable virtual body ownership was a predictor of participants’ change in compassion (*β*=0.29, 2-tailed t_59_=2.15, η_p_^2^=0.08, 95% CI 0.00-0.23; *P*=.04) and the predictor variable spirituality was a predictor of participants’ change in compassion (*β*=–0.35, 2-tailed t_59_=–2.89, η_p_^2^=0.13, 95% CI 0.01-0.30; *P*=.005), the feeling of presence was not a significant predictor (*β*=0.08, 2-tailed t_59_=0.6, η_p_^2^=0.001, 95% CI 0.00-0.10; *P*=.55). This means that the changes in compassion were predicted by participants’ spirituality and perceived level of virtual body ownership with moderate to large effects, but not by the perceived feeling of presence (Figure 5).

## Discussion

### Principal Findings

The study results indicate that engaging with the iVR nature embodiment scenario led to an increase in participants’ sense of nature connectedness, as well as a rise in compassion levels across all groups, despite no corresponding change in empathy, and no differential effects between groups (hypotheses 1, 2, and 3). Regarding the effect on symptom load (hypothesis 4), participants reported a reduction in subjective symptom load, as measured by VAS. Complementing these self-reported data, exploratory physiological measures via EDA revealed consistent arousal during the iVR application (hypothesis 5), highlighting that the immersive environment elicited a substantial affective response. Finally, changes in compassion were influenced by individual differences in spirituality and the degree of perceived virtual body ownership (hypothesis 6). Together, these results add further support to the multifaceted effects of iVR interventions on emotional and physiological states.

In general, the investigation of iVR nature exposure on symptom load was an important aim of this study. Here, we could show a significant decrease in individual symptom load in both patient groups, independent of previous symptom burden in the overall model, but significantly influenced by 2 covariates. The significant influence of the 2 PANSS factors, excitement and negative symptoms, calculated according to van der Gaag et al [34], is notable. In view of the known relationship of negative symptoms with poor treatment response [37], this positive association between negative symptoms and change in symptom load gives hope for a potentially effective intervention. Response of different symptom groups to comparable iVR interventions and individual characteristics influencing this response should be further studied, as it is still an insufficiently studied but promising field. Besides the above-mentioned study by Riches et al [16] with n=42 who found a decrease in VAS-measured stress, anxiety, and sadness [16], Ilioudi et al [38] conducted a quasi-randomized cross-sectional trial with 60 patients and compared the effects of iVR and physical calm rooms in acute psychiatric settings. Similar to our study, the authors not only use self-report questionnaires but also physiological parameters (blood pressure and heart rate). Of the 60 patients, 40 used the iVR environment, and 20 entered the physical calm room. Both groups showed an improvement in well-being, measured by VAS, after the intervention without showing significant differences between the 2 options [38]. Considering the potential benefits of iVR, as a cost-effective and space-saving tool, this study gives hope for an easily accessible nondrug therapeutic option. In a second study by the authors, this gratefulness of patients about nonpharmacological options was an important result, speaking for the good acceptance of such approaches [15]. In a longitudinal study over 3-4 weeks, Browning et al [39] examined the effect of daily iVR nature experiences on anxiety, depression, and rumination (n=24 in the iVR intervention group vs n=16 in the control group); however, not in a clinical cohort but in college students. Symptoms of anxious arousal and anxious apprehension decreased, whereas the VR nature experience did not influence anhedonia or rumination, thereby underlining the importance of specifying different symptom groups in a longitudinal setting [39].

Additionally, we could replicate findings from our earlier investigations [13,17] showing that using the *Tree* application fosters nature connectedness assessed with the INS scale. With this study, more than 200 participants tested the application, and in all studies, a significant increase in INS was found [13,17]. We also replicated the finding of an increase in compassion after exposure found in Spangenberger et al [17], as well as the perceived virtual body ownership of participants being a predictor for compassion. As an embodiment of a tree was an integral component of the used iVR scenario (embodying a tree) and a predictor of the changes in compassion, its role should be further studied with a focus on its potential in body psychotherapy, an established approach in the treatment of severe mental disorders [40]. Psychotic negative symptoms in particular are sometimes understood as a form of disembodiment and alienation from the self and are therefore addressed in body-oriented therapies [41]. Even though the relationship between nature embodiment, especially the feeling of growing virtually in this study, and specific symptoms or symptom load in the long term has not yet been sufficiently researched, the promising results from previous studies and this study provide grounds for more detailed investigations.

In this study, we also assessed empathy and observed that it is rather compassion than emotional or cognitive empathy that is elicited by the perceived virtual body ownership experience [17]. Again, we were able to support earlier findings that while compassion has some overlaps with the concept of empathic concern, it is recognized as a distinct emotion, separate from empathy, and is associated with activation in different brain regions [42-44]. Although different impairments of empathy are described in the 2 examined disorders [18,19], we could not find any differences regarding the change of empathy through the exposure between the 3 groups in our study.

### Limitations

Several limitations should be acknowledged when interpreting the findings of this study. First, our data were derived from an explorative study with a relatively small sample size, which restricts the generalizability of our results. Thus, further research with larger sample sizes is needed to assess the effects of iVR exposure on emotional or clinical aspects, for example, different categories of individual symptoms. The groups in this study were too small for the latter. Second, singular interventions do not allow for drawing conclusions about the long-term impact of this intervention. Longitudinal studies are necessary to clarify whether these effects change over time. Third, we did not ask participants’ previous iVR experience, which may influence observed outcomes. Fourth, we did not include a control group (eg, a different VR scenario) to differentiate between the effects of nature embodiment in iVR and the general effects of iVR, which should be supplemented in future studies. Fifth, the absence of systematic psychological side effect monitoring is due to the exploratory design of this study, but should be addressed in the following studies. Finally, the quality of EDA data collected was limited, likely due to issues related to the lack of a pre-VR baseline, sensor placement, signal noise, or participants’ movement. Even though the decision to not integrate a pre-VR baseline measure of EDA was made in order to limit the time commitment of the patients in this explorative study, future studies should integrate a pre-exposure baseline and separate tonic and phasic activity as recommended [45,46]. Larger samples might be essential for obtaining more reliable physiological measures.

### Conclusions

To conclude, the experience of embodying a tree in iVR had immediate benefits, as indicated by positive effects on individual symptoms, an increase in participants’ sense of nature connectedness, and a rise in compassion levels. It can be highlighted that patients with psychiatric conditions during inpatient treatment, including patients diagnosed with psychosis, were able to receive an iVR-based intervention and profited from the experience. Despite a still high technological effort to implement VR applications into therapeutic sessions, our study suggests that even a short exposure to becoming a virtual tree can elicit a strong affective response in patients. Given that some clinical facilities in urban areas offer little opportunity for recreation in natural surroundings, and that patients with acute psychiatric disorders are sometimes not able to seek out these surroundings on their own, the use of virtual nature experiences appears to be a promising approach. While these findings are a starting point, long-term effects in follow-up examinations remain to be investigated. Additional research examining sustained outcomes, different intervention dosages (longer or repeated sessions), sham or alternative VR control conditions, as well as the integration of such virtual embodiment practices into established body therapy interventions, would help determine whether this innovative form of iVR exposure can become an effective and easily accessible nonpharmacological tool for patients with severe mental disorders.

## Data Availability

The source data used in this research are currently unavailable for public sharing due to strict data safety and confidentiality protocols mandated by our university. These restrictions are in place to ensure compliance with ethical standards, privacy regulations, and institutional policies. Access to the data may be granted under specific circumstances, subject to appropriate data use agreements and ethical approvals. For more information about the data, please contact the corresponding author.

## Appendix

Multimedia Appendix 1 Additional information on statistical analysis.

## Abbreviations

BDI: Beck Depression Inventory
DSM-IV: Diagnostic and Statistical Manual of Mental Disorders (Fourth Edition)
EDA: electrodermal activity
HC: healthy controls
HMD: head-mounted display
INS: Inclusion of Nature in Self
iVR: immersive virtual reality
MET: Multifaceted Empathy Test
NS-SCR: nonspecific skin conductance response
PANAS: Positive and Negative Affect Schedule
PANSS: Positive and Negative Syndrome Scale
SCID I: Structured Clinical Interview for DSM-IV (Diagnostic and Statistical Manual of Mental Disorders, Fourth Edition)
VAS: visual analog scale
VR: virtual reality
