# Supplementary material for: Effects of Virtual Nature Embodiment on Compassion, Empathy, Nature Connectedness, and Symptom Burden in Patients With Psychosis or Depression: An Explorative Clinical Study
Source: JMIR Serious Games. 2026 Mar 24;14:e74337. doi: 10.2196/74337 (PMC13058528; doi:10.2196/74337)
Supplement: Multimedia Appendix 1 [file games_v14i1e74337_app1.docx]

Supplementary material

**Statistics**

H1

The dependent variable nature connectedness was normally distributed in each group assessed with the Shapiro-Wilk-test and QQ-plots. Homogeneity of variances was asserted using Levene’s Test which showed that equal variances could be assumed. There were no outliers in the data (using box-plots).

H2

To analyze H2, the subscales for Cognitive Empathy and Emotional Empathy Explicit as dependent variables of both sequences (A and B) were first examined for normal distribution and then, since they were normally distributed, examined for differences in mean values using t-tests for independent samples. Sequences A and B were then treated as not significantly different. Repeated measures ANOVA were calculated to test for significant differences between Cognitive Empathy and Emotional Empathy Explicit pre vs. post VR-exposure in the three groups. Outliers were included in the analyses.


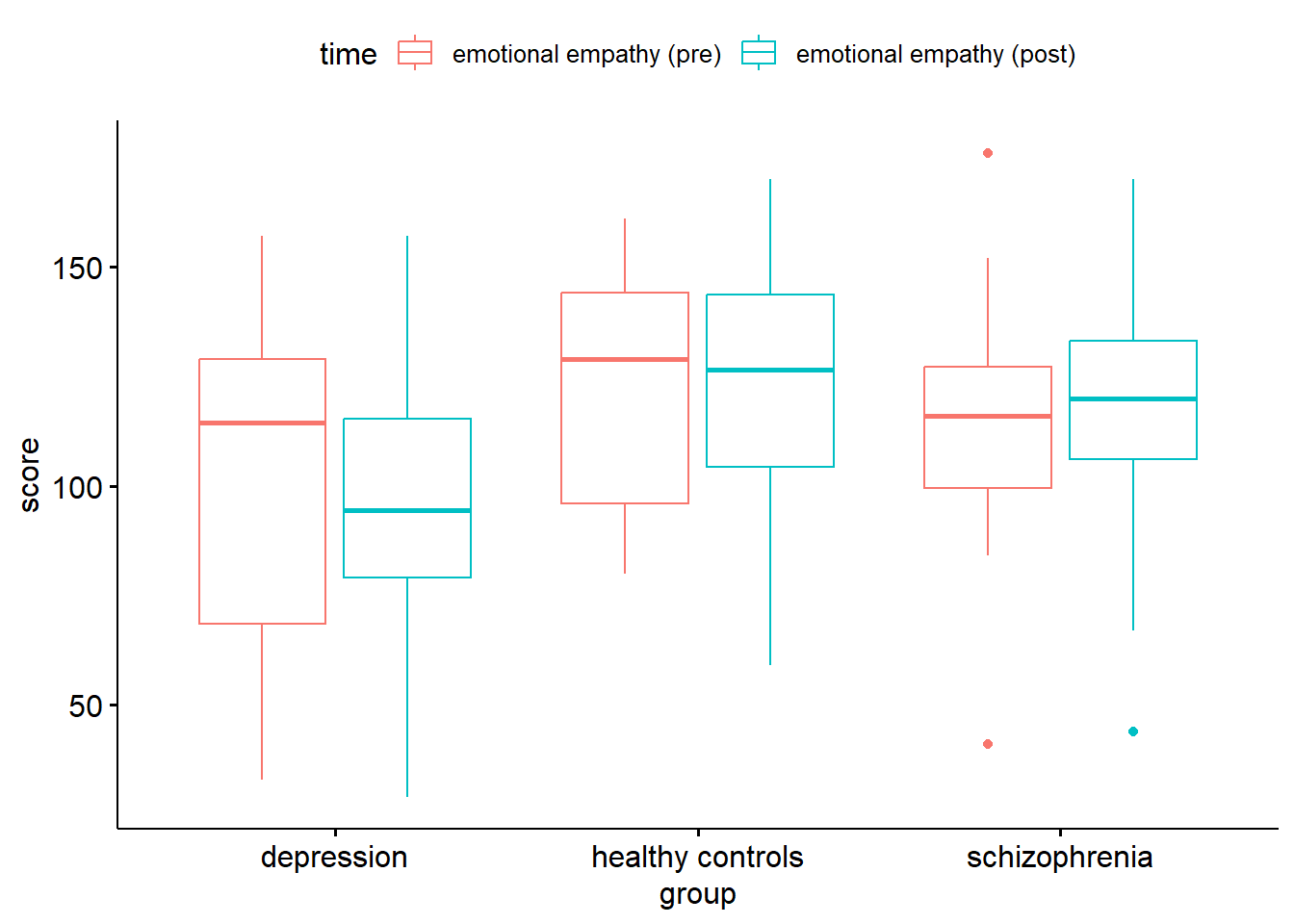


Fig 1: Emotional Empathy pre and post VR


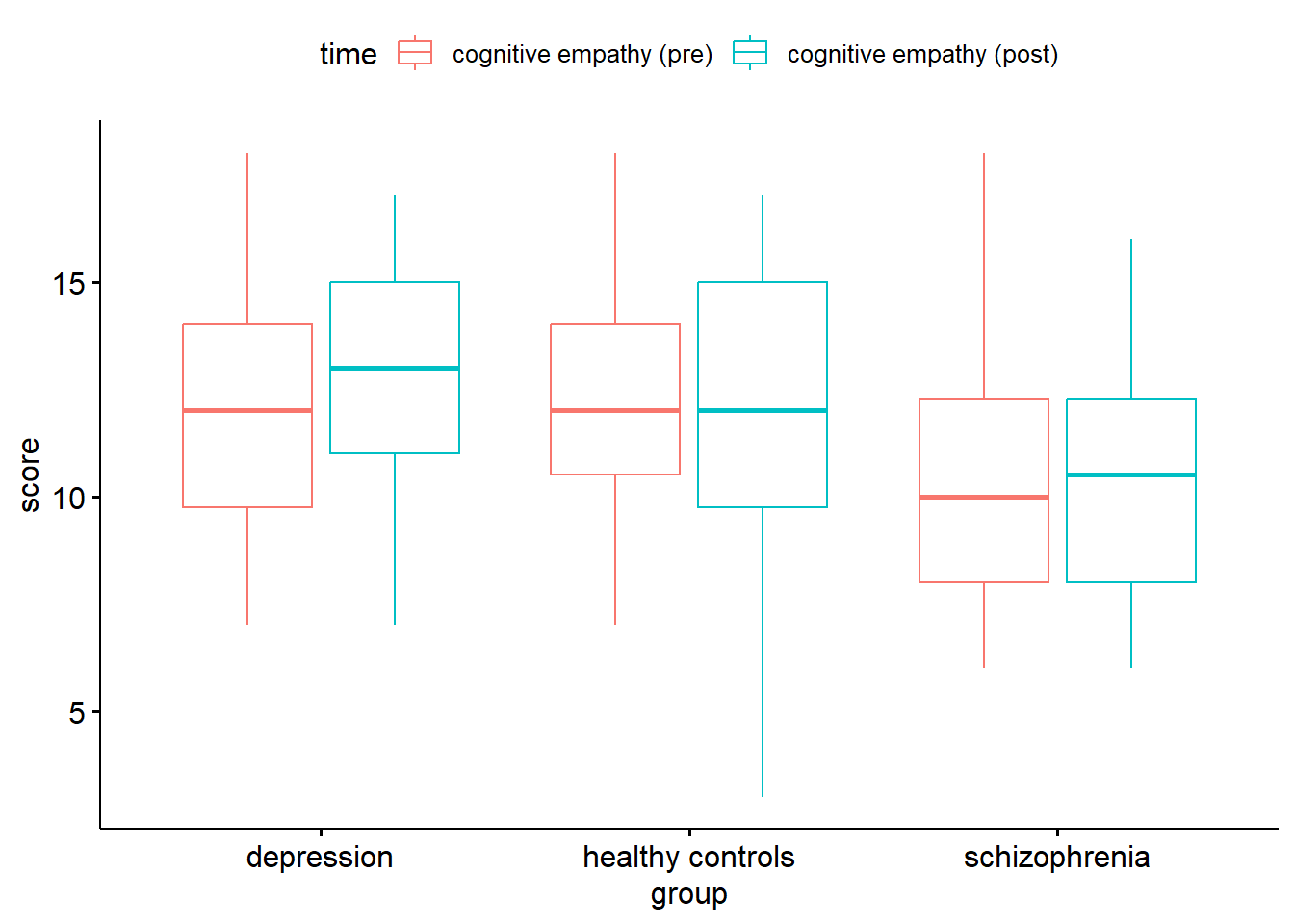


Fig 2: Cognitive Empathy pre and post VR

H3

To analyze H3 we calculated a one-way repeated measures analysis of variance (ANOVA), and if means were statistically significant, we calculated post-hoc tests. The dependent variable compassion was normally distributed before and after the experiment assessed with the Shapiro-Wilk-test. Homogeneity of variances was asserted using Levene’s Test which showed that equal variances could be assumed. There were three moderate outliers in the data. Results of the ANOVA didn’t vary significantly when comparing analysis with and without outliers.


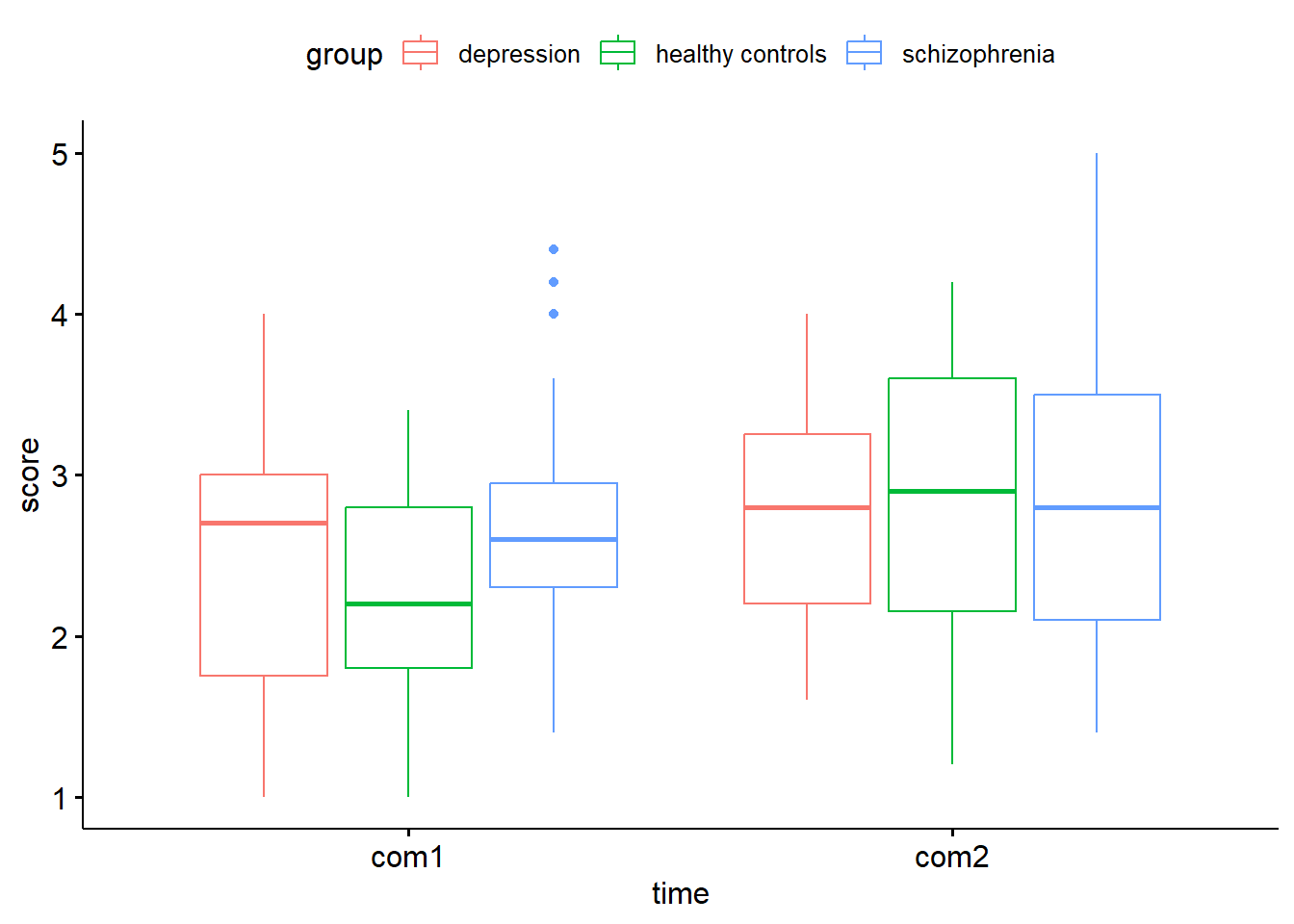


Fig.3: Compassion pre (com1) and post (com2) VR

H4

To analyze H4 we first calculated the mean of the VAS values before and after the VR intervention and tested them for normal distribution. Shapiro Wilk test showed a violation of normal distribution in the group of depressive patients, Kolmogorov-Smirnov tests and QQ plots however affirmed a normal distribution of VAS mean values. Then, repeated measures ANOVA were conducted to test for significant differences in mean VAS scores pre vs. post VR exposure in the two patient groups. The analysis was conducted with and without one moderate outlier, showing no significant differences. Additionally, we conducted ANCOVA with depressive (BDI sum score) and psychotic symptom severity (PANSS subscales according to the five-factor solution by van der Gaag et al.) as covariates and delta VAS as the dependent variable.

H6

Before conducting multiple regression analyses, we assessed normality with the Shapiro-Wilk test:

Spirituality was normally distributed for depressive or psychotic participants, but not for healthy participants, as assessed by the Shapiro-Wilk test (α = .05).

Presence was normally distributed for the three groups as confirmed by the Shapiro-Wilk test. For the variable embodiment in VR, the Shapiro-Wilk test showed a violation of normality for the schizophrenia group (*p* = .043) whereas values of the depression group and healthy controls were normally distributed. Four outliers were identified via boxplots of presence values in the group of healthy controls. Six outliers were identified in the visualization of embodiment values. All outliers were included in the regression analyses.

**Additional Analyses**

**Positive and Negative Affect Schedule (PANAS)**

In the patient group, negative affect (NA) significantly decreased from pre-test (M = 1.84, *SD* = 0.83) to post-test (M = 1.43, *SD* = 0.43), t(39) = 4.74, p < .001.

Similarly, the control group showed a smaller but still significant reduction in negative affect, t(19) = 3.20, p = .005, with means decreasing from M = 1.28 (*SD* = 0.36) to M = 1.18 (*SD* = 0.33).
